# Supra-Physiological Levels of Magnesium Counteract the Inhibitory Effect of Zoledronate on RANKL-Dependent Osteoclastogenesis

**DOI:** 10.3390/biology14050533

**Published:** 2025-05-11

**Authors:** Lorenzo Caselli, Lisa De Pasquale, Rossella Palumbo, Silvia Ricchiuto, Monica Montanari, Sebastiano Rontauroli, Alessandra Ottani, Ruggiero Norfo, Tommaso Zanocco-Marani, Alexis Grande

**Affiliations:** 1 Department of Biomedical, Metabolic and Neural Sciences, University of Modena and Reggio Emilia, 41125 Modena, Italy; 284169@studenti.unimore.it (L.C.); 177443@studenti.unimore.it (L.D.P.); 255698@studenti.unimore.it (S.R.); alessandra.ottani@unimore.it (A.O.);; 2 Department of Life Sciences, University of Modena and Reggio Emilia, 41125 Modena, Italy; monica.montanari@unimore.it

**Keywords:** osteoclasts, magnesium, bisphosphonates, zoledronate, denosumab, ONJ

## Abstract

Bisphosphonates (BPs) are drugs used to cure metabolic diseases like osteoporosis and oncological conditions, such as multiple myeloma and bone metastases. The pharmacological activity of these compounds is mediated by their capacity to induce a systemic osteoclast depletion, finally resulting in reduced bone resorption. In spite of their efficacy, the clinical application of BPs is sometimes associated with a frightening side effect known as osteonecrosis of the jaw (ONJ). In principle, a therapeutic approach able to elicit the local re-activation of osteoclast production could counteract the onset of ONJ and promote the healing of its lesions. Using a vitamin D3-dependent model of osteoclast differentiation, it has been previously demonstrated that when used at supra-physiological concentrations, magnesium strongly favors the process under consideration, and its effect is furtherly enhanced by the presence of a BP called zoledronate. Here, we show that similar results can be obtained in a RANKL-dependent model of osteoclast differentiation, suggesting that a topical therapy based on magnesium may be also suitable for ONJ determined by denosumab in light of the ability of this monoclonal antibody to target RANKL.

## Simple Summary

Bisphosphonates (BPs) are drugs used to cure metabolic diseases like osteoporosis and several cancers, such as multiple myeloma and bone metastases. They exert their effect by reducing the activity of osteoclasts that are cells responsible for bone destruction. For this reason, they are classified among anti-resorptive agents. Unfortunately, in spite of their undisputed efficacy, these drugs can cause a dangerous side effect called osteonecrosis of the jaw (ONJ). Researchers have been exploring different ways to reverse ONJ by encouraging the activity of osteoclasts in the jaw, which could help heal the bone damage. Previous reports have shown that high levels of magnesium can favor the development of osteoclasts promoted by vitamin D3, and this effect is even stronger in the presence of a BP named zoledronate. In this study, we found that magnesium also favors osteoclast formation in the presence of a more important osteoclastogenic factor called RANKL. This observation suggests that magnesium could help treating ONJ even when this condition is provoked by a distinct anti-resorptive agent named denosumab, which is a monoclonal antibody-neutralizing RANKL.

## 1. Introduction

Bone, a dynamic and multifunctional tissue, serves as a structural framework for the human body, facilitates mobility, and protects vital organs. Beyond its mechanical functions, bones play a pivotal role in mineral homeostasis, hematopoiesis, and endocrine regulation [1]. Throughout life, bones are subjected to a continuous remodeling, a balanced process of resorption and formation, respectively, mediated by osteoclasts and osteoblasts [2]. Disruptions of this delicate balance or the emergence of malignancies within bone tissue can lead to a spectrum of disorders with profound consequences for skeletal health and overall well-being [3].

These conditions are broadly categorized into metabolic and oncologic bone diseases. Metabolic bone diseases encompass conditions that impair normal bone mineralization and remodeling [4]. These disorders often arise from systemic factors such as hormonal imbalances, nutritional deficiencies, or genetic abnormalities. Among the most prevalent of these conditions is osteoporosis, characterized by reduced bone mass and micro-architectural integrity. This condition is followed by Paget’s disease, caused by accelerated bone turn-over, and osteomalacia, which, together with its pediatric counterpart, is called rickets, resulting from defective bone mineralization induced by vitamin D3 or phosphate deficiency. Typical features of metabolic bone diseases are represented by chronic pain, fractures, and deformities, limiting mobility, and creating disability and a diminished quality of life. Oncologic bone diseases include primary bone tumors, metastatic lesions, and hematologic malignancies affecting bone. While primary bone cancers, such as osteosarcoma and Ewing sarcoma, are rare, their aggressive nature and predilection for younger individuals highlight their clinical significance [5]. In contrast, bone metastases are among the most common oncologic complications, particularly in cancers originating from the breast, prostate, lung, and kidney [6]. These metastatic lesions often disrupt bone homeostasis, leading to pathological fractures, severe pain, and metabolic complications like hypercalcemia. Similarly, multiple myeloma, a malignancy of plasma cells, causes osteolytic lesions and systemic effects such as anemia and renal dysfunction [7,8].

Anti-resorptive drugs, particularly bisphosphonates (BPs) and denosumab (DMab), have become a cornerstone in managing these conditions [9,10]. BPs, the most widely used class of such compounds, are synthetic analogues of pyrophosphate with a high affinity for hydroxyapatite. This feature allows them to localize in bone, where they induce osteoclast apoptosis and, to a lesser extent, inhibit their differentiation. In cancer-like conditions such as breast and prostate cancer, as well as multiple myeloma, BPs are used to manage bone metastases and prevent skeletal-related events (SREs) such as fractures, spinal cord compression and hypercalcemia [11]. Zoledronate and pamidronate, belonging to the so called nitrogenous-bound category of BPs, have been frequently utilized for this purpose [12]. While BPs offer substantial therapeutic benefits, their use is not without challenges, with osteonecrosis of the jaw (ONJ) being a notable and severe adverse effect [13,14,15]. This condition is defined as an exposed necrotic bone in the maxillo-facial region persisting for more than eight weeks in patients with a history of anti-resorptive therapy, without prior radiation therapy to the jaw. The exact mechanism underlying ONJ remains unclear, but several hypotheses have been proposed. BPs suppress osteoclast activity, leading to a reduction in bone turn-over, highly required in the jawbone [16,17,18]. In addition, while beneficial in contrasting systemic bone loss or destruction, this suppression may impair the ability of the interested site to heal after trauma or surgery. In this regard, local infection, often following dental extractions or poor oral hygiene, may exacerbate ONJ [19,20]. Finally, BPs inhibit farnesyl–pyrophosphate synthase (FPPS), a key enzyme in the mevalonate pathway. This inhibition also affects endothelial cell function, potentially impairing angiogenesis and reducing vascular supply to the jawbone [21]. DMab is a monoclonal antibody able to inhibit osteoclast formation by inactivating its main factor called RANKL (receptor activator of nuclear factor-kappa B ligand). Unsurprisingly, ONJ, initially ascribed to BPs, has been subsequently also associated to DMab use [22], indicating that it is a common complication of all anti-resorptive therapies [23,24]. ONJ management aims to alleviate symptoms, prevent progression, and promote healing while minimizing invasive interventions [25,26,27,28].

Recently, different experimental approaches aimed to restore osteoclast differentiation and activity, thereby improving bone formation and remodeling in ONJ, have been proposed [29]. They are based on the following: administration of teriparatide, a parathyroid hormone analog [30]; inhibition of glycogen synthase kinase-3 β (GSK-3β), for example, with lithium chloride (LiCl) [31]; administration of geranyl–geraniol [32]; and local use of magnesium [33]. Magnesium plays a dual role in osteoclast differentiation, with its effects varying depending on the used concentration (infra- versus supra-physiological), chemical form (metallic versus salts), and treated organism (humans versus animals). While there is a general agreement that magnesium deficiency leads to osteoclast activation [34,35,36], the effects determined by magnesium excess are more controversial and influenced by various factors, particularly the adopted formulation (metal or salt) and the considered species (rodents or humans). Several studies have shown that hyper-stimulation with magnesium inhibits osteoclast function in rats [37,38], whereas in humans, it has been observed to promote activation [39,40]. Additionally, metallic magnesium has demonstrated an ability to inhibit osteoclastogenesis [41,42], while magnesium salts tend to enhance it [39,40]. These opposing effects have also been confirmed within the context of a single study, where the two magnesium formulations were incubated with cultured osteoclasts derived from human monocytes [43]. Experiments performed in our laboratory have demonstrated that supra-physiological concentrations of magnesium promote osteoclast differentiation in a vitamin D3-dependent model of human osteoclastogenesis based on the U937 cell line [33,39,40]. These data also showed that the presence of zoledronate further enhances the capacity of magnesium to induce the studied process [33]. In this regard, it is important to distinguish hyper-stimulation with magnesium (exposure to supra-physiological concentrations) from treatments addressing its deficiency. The latter, in our opinion, should be considered as a normalization (or rescue) of the deficiency state, eliciting an apparent (or relative) inhibition of osteoclast activity due to the cessation of deficiency-induced activation [44,45]. Similarly, studies detecting reduced bone turn-over upon dietary magnesium supplementation in humans [46,47], besides their contradictory conclusions [48,49,50,51,52], actually operate in a physiological range of concentrations (next to 1 mM). These conditions are not comparable to what happens in hyper-stimulation, evaluating instead the effects of up to 10-times higher concentrations of magnesium (around 10 mM).

In this paper, we assessed the ability of supra-physiological concentrations of magnesium to support osteoclast differentiation in a RANKL-dependent model of human osteoclastogenesis based on the THP-1 cell line [53]. The data obtained confirmed that magnesium is capable of hindering the inhibitory effect exerted by zoledronate on osteoclast differentiation. At the same time, the RANKL-dependent nature of the experimental model used to conduct our study indicates that a local therapy with magnesium might be effective also in the treatment of DMab-induced ONJ.

## 2. Materials and Methods

### 2.1. Cell Culture

The THP-1 cell line was provided by the American Type Culture Collection (TIB-202, ATCC; Rockville, MD, USA) and cultured at 37 °C, 5% CO_2_, in an RPMI1640 medium (Euroclone, Devon, UK), supplemented with 10% heat-inactivated fetal bovine serum (FBS) (Sigma-Aldrich, St. Louis, MO, USA), 1 mM L-glutamine, and 1× penicillin/streptomycin (Euroclone, Pero, Italy).

Zoledronate (Zoledronic Acid, ZA) and magnesium chloride (MgCl_2_) (Sigma-Aldrich, St. Louis, MO, USA) were dissolved in sterile water and added to cell cultures at concentrations that are indicated in the Results.

Osteoclast differentiation of THP-1 cells was induced by a 2-day stimulation with 48 nM phorbol 12-myristate 13-acetate (PMA) (Sigma-Aldrich, St. Louis, MO, USA), followed by a rinse with pure RPMI1640, aimed to eliminate non-adhering undifferentiated cells and residual PMA, and then by a 1- to 2-week stimulation with 25 ng/mL human monocyte-colony stimulating factor (M-CSF) and RANKL (Miltenyi Biotec, Auburn, CA, USA).

The effects of ZA and MgCl_2_ on THP-1-derived osteoclasts were assessed by molecular, flow cytometry, microscopic, and cytochemical analysis, all described in detail below.

### 2.2. Molecular Analysis

Total RNA was extracted using the Qiagen RNeasy plus mini kit (Qiagen, Valencia, CA, USA) and quantified with a NanoDrop 1000 spectrophotometer (Thermo Fisher Scientific, Waltham, MA, USA). Messenger RNA expression was analyzed by a quantitative real-time polymerase chain reaction (QRT-PCR) method based on the Taqman principle. For this purpose, 200 ng of each RNA sample was subjected to reverse transcription (RT) using the HiScript III RT SuperMix for qPCR (Vazyme Biotech, Nanjing, China). Amplification was performed adding to the cDNA sample the Taqman Gene Expression Master Mix (Thermo Fisher Scientific, Waltham, MA, USA) and the Taqman Gene Expression Assays (Thermo Fisher Scientific, Waltham, MA, USA), listed below, together with their gene symbol and catalogue numbers: receptor activator of NF-kB (RANK, Hs00921372_m1), nuclear factor of activated T cells 1 (NFATC1; Hs00542678_m1), acid phosphatase (ACP5, also known as Tartrate Resistant Acidic Phosphatase or TRAP; Hs00356261_m1), cathepsin K (CTSK; Hs00166156_m1), Matrix Metallo Proteinase 9 (MMP9; Hs00234579_m1), Musculo–Aponeurotic Fibrosarcoma oncogene homolog B (MAFB; Hs00534343_s1), Cluster Designation 14 (CD14; Hs02621496_s1), Cluster Designation 163 (CD163; Hs00174705_m1), cyclin-dependent kinase inhibitor 1 A (CDKN1A, also known as p21; Hs00355782_m1), and glyceraldehyde-3-dehydrogenase (GAPDH; Hs02758991_g1). Once prepared, QRT-PCR reactions were carried out in triplicate in a Light Cycler 480 thermal cycler (Roche Diagnostics, Mannheim, Germany), which measures the mRNA expression of each analyzed gene in the considered sample, in comparison with a control sample, upon normalization with a constitutive gene, represented in our procedure by the GAPDH gene. This operation gives rise to a parameter named relative quantity (RQ), that, in our context, was used to indicate the extent of mRNA level variations elicited by the various analyzed treatments.

### 2.3. Flow Cytometry Analysis

This analysis was performed with an Attune NxT flow cytometer (Thermo Fisher Scientific, Waltham, MA, USA) to assess the cell cycle, apoptosis, and differentiation effects determined by the studied compounds.

Cell cycle and apoptosis were estimated by mono-parametric flow cytometry, carried out upon a 30 min incubation at 4 °C with Nicoletti’s solution, containing 20 µg/mL propidium iodide (PI) 0.1% triton X–100, and 0.1% tri-sodium citrate, all provided by Sigma-Aldrich, St. Louis, MO, USA, and dissolved in distilled water.

The entity of differentiation was evaluated after an incubation for 30 min at 4 °C, in PBS containing 5% FCS and 1% FcR blocking reagent (Miltenyi Biotec, Auburn, CA, USA), in the presence of fluorescein isothiocyanate-conjugated (FITC) mouse anti-human CD14 monoclonal antibody (MoAb) and phycoerythrin-conjugated (PE) mouse anti-human CD11b MoAb (Miltenyi Biotec, Auburn, CA, USA), both diluted at a 1:100 ratio. At the end of labeling, cells were washed and re-suspended with PBS.

### 2.4. Cell Visualization

Live images of cell samples under various analyzed culture conditions were obtained using the digital microscope Evos M500 Imaging System (Thermo Fisher Scientific, Waltham, MA, USA).

Cytochemical analysis of TRAP activity was carried out on cyto-centrifuged slides using the TRAP staining kit (Cosmo Bio, Tokyo, Japan). To this aim, one tenth of a 1 mL cell suspension of each sample, cultured in a multi 24-plate well previously seeded with 5 × 10^5^ cells, was loaded on a corresponding cytospin. At the end of the reaction, stained cells were counted by microscope examination conducted at a 400× magnification on a total number of 500 cells. The results obtained were finally presented as a percentage of cell positivity and number of positive cells/mm^2^.

### 2.5. Statistical Analysis

All experiments were repeated at least three times and the results are presented as the mean ± S.E.M. values. Statistical analysis was performed with the ANOVA procedure, followed by post hoc pairwise comparisons carried out according to the Bonferroni method. Both tests were executed using the Prism 10.4.2 software. Data arising from this analysis were considered significant when exhibiting *p*-values ≤ 0.05 and highly statistically significant when exhibiting *p*-values ≤ 0.01.

## 3. Results

### 3.1. Effects Determined by Treatment with MgCl_2_ and ZA on the Osteoclast Differentiation of THP-1 Cells, Induced by Stimulation with Phorbol Esters, M-CSF, and RANKL

**Figure 1:**
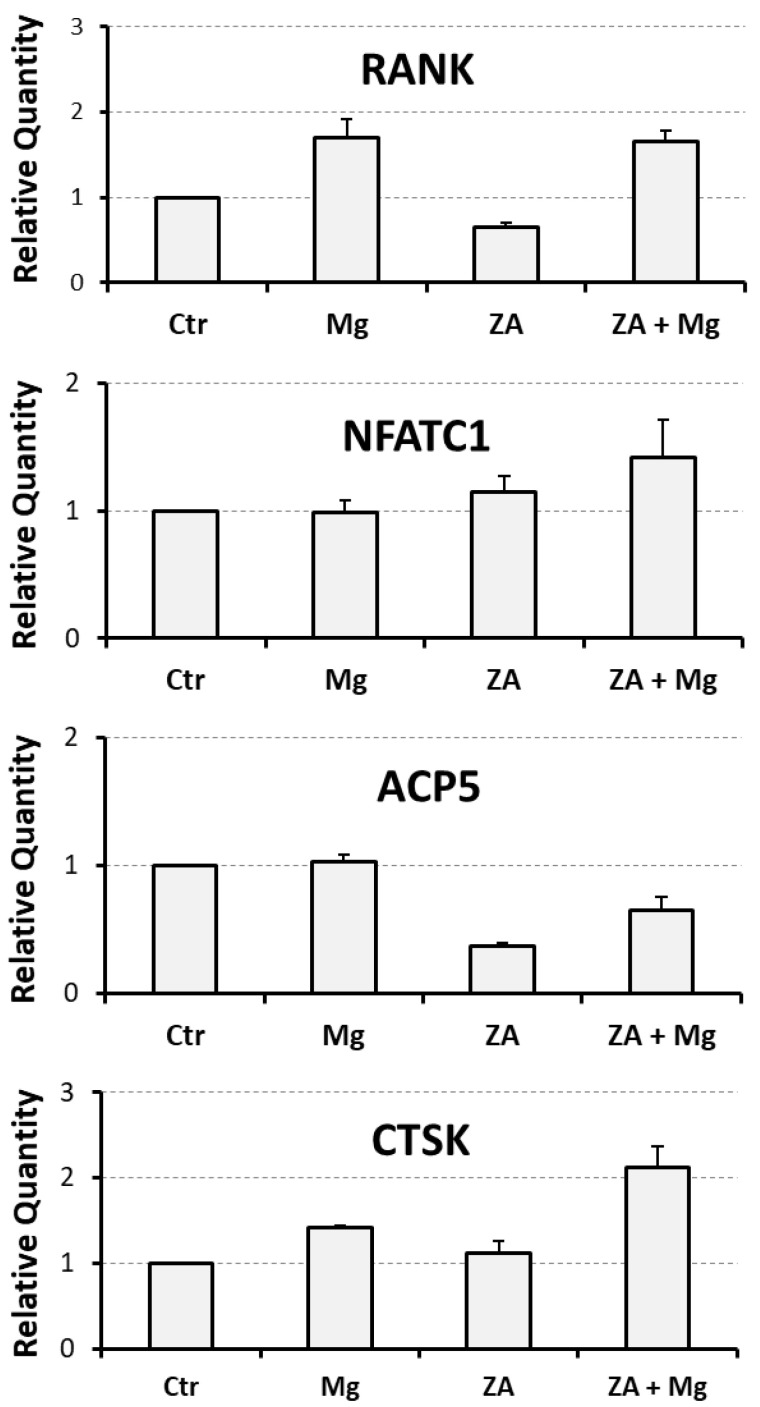
Molecular analysis of osteoclast markers in THP-1-derived osteoclasts under treatment with ZA and MgCl_2_ used alone or in combination. Transcript levels of osteoclast markers were analyzed by QRT-PCR in THP-1 cells differentiated to osteoclasts upon 2-day incubation with PMA, followed by a 7-day stimulation with M-CSF and RANKL. Contextually, these cells were also treated with zoledronate (ZA), MgCl_2_ (Mg), both compounds (ZA + Mg) or neither of the two (Ctr). The results are presented as bar histograms in which the values of relative quantity are reported on the y-axis and tested treatments are indicated on the x-axis. Data are shown as the mean ± S.E.M. of four independent experiments. Analyzed genes are indicated on the top of each histogram.

**Figure 2:**
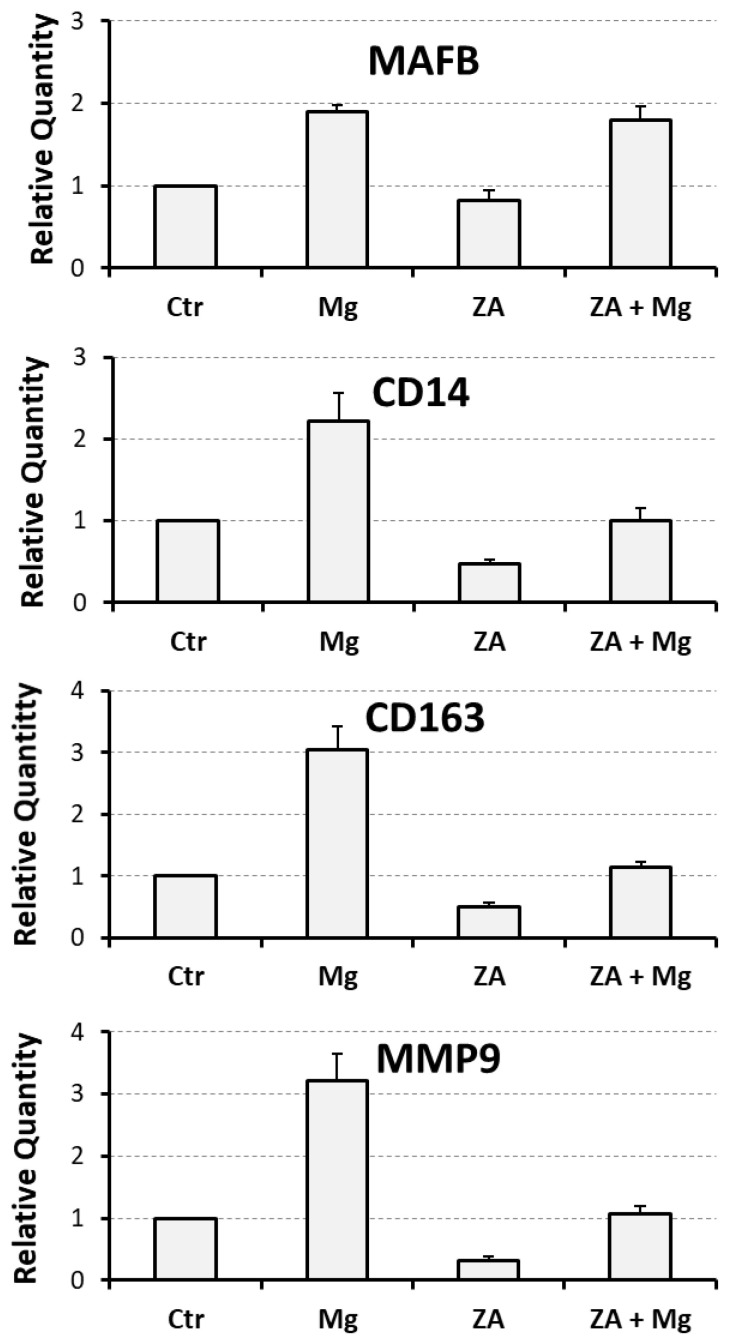
Molecular analysis of monocyte–macrophage markers in THP-1-derived osteoclasts under treatment with ZA and MgCl_2_ used alone or in combination. Transcript levels of monocyte–macrophage markers were analyzed by QRT-PCR on the same cell samples of Figure 1. The data obtained are presented as described therein, using the same acronyms to indicate the analyzed treatments.

To evaluate the capacity of MgCl_2_ and ZA to affect osteoclast differentiation, we utilized an experimental model of RANKL-dependent osteoclastogenesis, represented by THP-1 cells subjected to an initial 2-day stimulation with PMA followed by a 7-day stimulation with M-CSF and RANKL. Under these culture conditions, cells were treated with 10 mM MgCl_2_ and 10 µM ZA, which were added alone or in combination. Cells differentiated with the same modalities, but untreated with MgCl_2_ and ZA, were used as a control. The choice of the mentioned concentrations was based on our experience and the data available in the scientific literature, and it has already been explained in detail in a previous report published by our research group [33]. Briefly, 10 mM, in comparison with lower amounts, was the concentration of MgCl_2_ determining the best osteoclastogenic effect in vitro [39]. Similarly, a 10 µM concentration of ZA, besides its optimal anti-osteoclast effect in vitro, has been adopted in the past by many authors working in the field, and is very close to the concentration reached in vivo, inside bone tissue in patients under treatment with this drug (ranging 0.5 to 5 µM) [33]. In addition, both concentrations are well tolerated in cell culture [33]. At the end of the experiment, all samples underwent molecular analysis, performed by QRT-PCR, to evaluate the mRNA expression levels of a number of osteoclast and monocyte–macrophage differentiation markers [33,39,40,54,55,56,57]. The osteoclast markers included the following: RANK, receptor of RANKL; NFATC1, the master regulator of osteoclast differentiation; ACP5, a phosphatase mediating the degradation of bone hydroxiapatite crystals; CTSK, a protease mediating the degradation of extra-cellular bone collagen; and MMP9, an enzyme endowed with a similar activity. The monocyte–macrophage markers included instead were as follows: MAFB, the master regulator of monocyte–macrophage differentiation; CD14, a surface antigen more related to monocytes and M1 activation; and CD163, a surface antigen more related to macrophages and M2 activation. The results of this analysis are presented in Figure 1 and Figure 2 as bar histograms, and in Table 1, as numerical values, always reporting the RQ values obtained in the various QRT-PCR reactions in terms of the mean ± S.E.M. It is worth considering that we preferred to present the MMP9 gene together with monocyte–macrophage markers for its particular capacity to mimic the CD163 mRNA expression pattern [33,58].

Treatment with MgCl_2_ promoted, compared to the control, an up-regulated mRNA expression of RANK and CTSK osteoclast differentiation markers, but not of NFATC1 and ACP5 (Figure 1). Among the monocyte–macrophage markers, MgCl_2_ treatment strongly induced the transcription of all the four analyzed genes, that is MAFB, CD14, CD163, and MMP9, where the last two were most up-regulated (Figure 2). Treatment with ZA, as expected, strongly inhibited the transcription of both osteoclast and monocyte–macrophage differentiation markers (Figure 1 and Figure 2). Combined treatment with ZA and MgCl_2_ rescued the inhibition observed by treatment with ZA alone, and restored the levels of all transcripts. In particular, RANK, NFATC1, and CTSK osteoclast markers returned to levels comparable to those observed by MgCl_2_ treatment, while ACP5, was up-regulated compared to treatment with ZA alone, but remained lower to what was observed by MgCl_2_ treatment alone (Figure 1). As far as monocye–macrophage markers are concerned, combined treatment with ZA and MgCl_2_ rescued the transcription of all four markers compared to treatment with ZA, but only the expression of MAFB returned to levels comparable to MgCl_2_ treatment (Figure 2). Statistical analysis, conducted with the ANOVA procedure, showed that with the only exception of NFATC1, all analyzed genes exhibited highly statistically significant variations in their mRNA expression levels (Table S1, Supplementary Material). The Bonferroni test put in evidence that three osteoclast markers (RANK, ACP5, CTSK) and one macrophage marker (MAFB) were highly statistically significant between cells treated with ZA + MgCl_2_ and cells treated with ZA alone.

### 3.2. Proliferative and Apoptotic Effects Determined by Treatment with MgCl_2_ and ZA During the Osteoclast Differentiation of THP-1 Cells, Induced by Stimulation with Phorbol Esters, M-CSF, and RANKL

To assess whether MgCl_2_ and ZA were also able to influence proliferation and apoptosis during the osteoclastogenesis process, THP-1 cells under the experimental conditions of the previous paragraph were subjected to flow cytometry analysis performed upon PI staining and QRT-PCR analysis aimed to estimate the mRNA expression of the p21 growth arrest gene.

**Figure 3:**
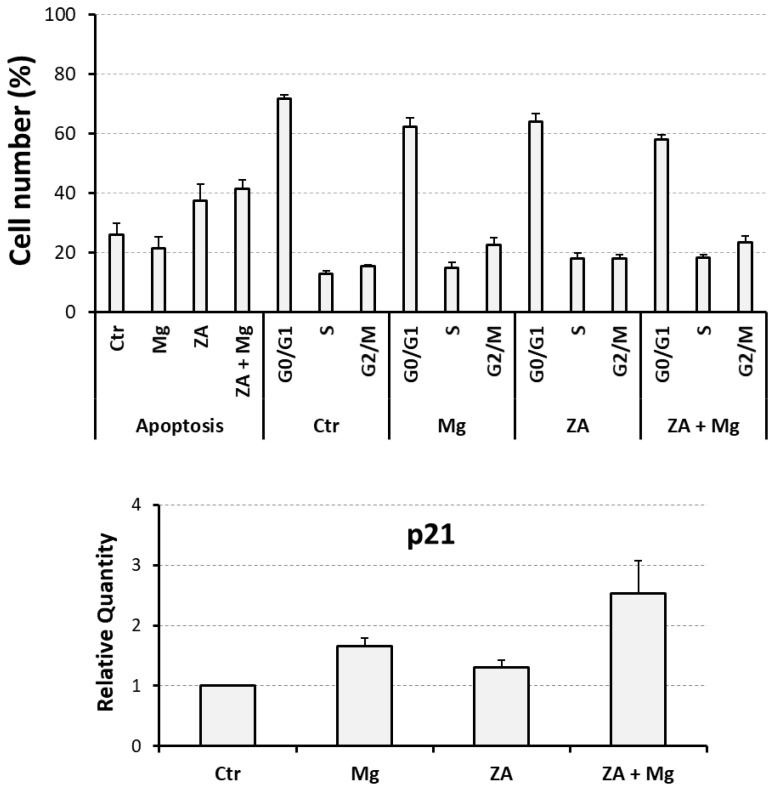
Flow cytometry analysis of apoptosis and cell cycle in THP1-derived osteoclasts under treatment with ZA and MgCl_2_ used alone or in combination. Apoptosis and cell cycle were analyzed in cell samples under the same experimental conditions of Figure 1 and Figure 2. Upper panel shows the number of apoptotic cells and the distribution in the various phases of cell cycle as assessed by flow cytometry analysis after staining with propidium iodide. Results are presented as a bar histogram indicating cell percentages on the *y*-axis and the tested treatments, or the analyzed parameters, on the *x*-axis. Lower panel shows the results of the QRT-PCR analysis of p21 mRNA expression performed and presented as already explained in the legend of Figure 1 and Figure 2. In both panels, data are presented as the mean ± S.E.M. values of four independent experiments.

The results obtained are shown in Figure 3 and Table 2, and indicate that, as expected, ZA determines an appreciable induction of apoptosis, but the addition of MgCl_2_ is not able to promote a relevant increase in this effect, suggesting that the combined treatment with ZA and MgCl_2_, is well tolerated by the analyzed cells. Treatment with MgCl_2_, either alone or in combination with ZA, also induced a slight increase in the G_2_/M peak of the cell cycle that, as indicated by the QRT-PCR analysis of p21 gene in the same cell populations, is concurrent with a proliferation arrest. This effect might depend on the ability of MgCl_2_ to regulate protein kinases in general, and possibly also cyclin–cyclin-dependent kinase (CDK) complexes controlling the M phase of cell cycle. Statistical analysis carried out with the ANOVA procedure showed that the effect exerted by the tested treatments was highly statistically significant on apoptosis and the G_2_/M phase of the cell cycle (Table S2, Supplementary Material), whereas it was simply statistically significant on p21 mRNA expression (Table S3, Supplementary Material). The Bonferroni test was statistically significant for apoptosis between ZA-treated and control cells, but not significant between ZA + MgCl_2_ treatment and ZA treatment, indicating, again, that MgCl_2_ does not exacerbate the apoptotic effect of ZA.

### 3.3. Effects Determined by Treatment with MgCl_2_ and ZA on the Expression of CD14 and CD11b Surface Antigens During the Osteoclast Differentiation of THP-1 Cells Induced by Stimulation with Phorbol Esters, M-CSF, and RANKL

**Figure 4:**
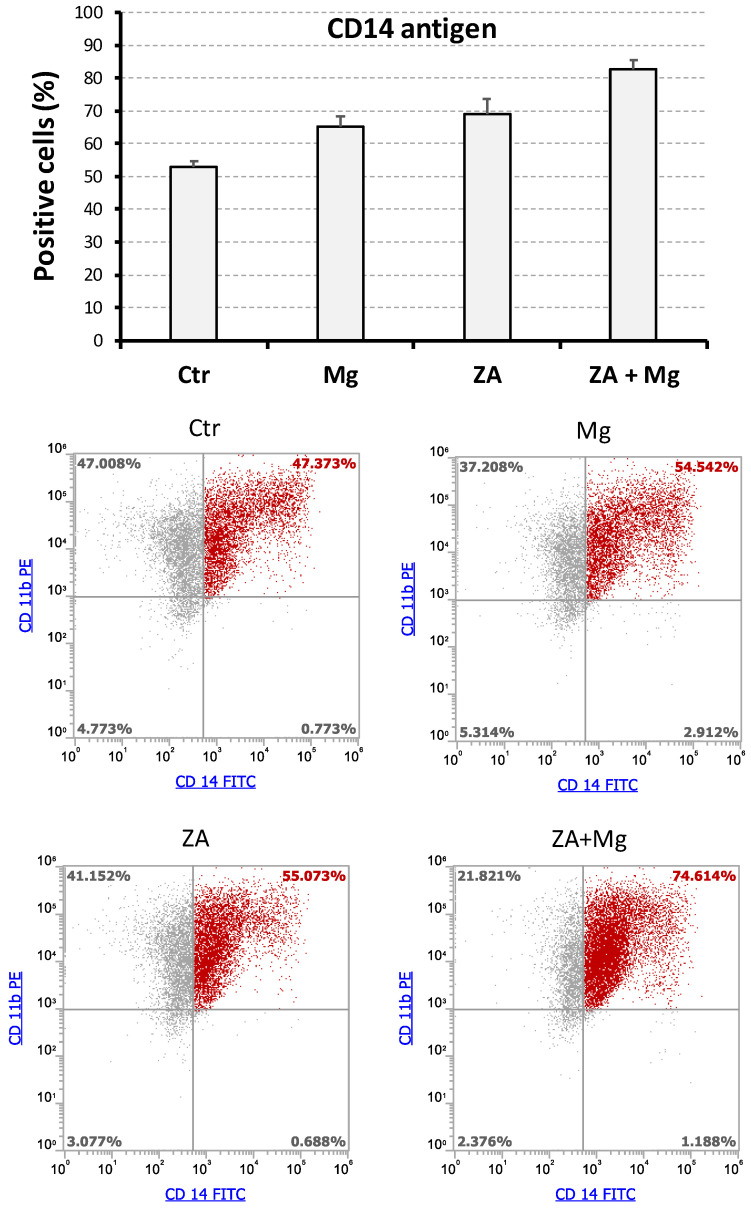
Flow cytometry analysis of surface antigens in THP-1-derived osteoclasts after a 7-day stimulation with M-CSF/RANKL and parallel treatment with ZA/MgCl_2_. Expression of the CD14 and CD11b surface antigens was assessed by the flow cytometry analysis of THP1 cells differentiated to osteoclasts upon a 2-day incubation with PMA followed by 7-day stimulation with M-CSF and RANKL. Contextually, these cells were also treated with zoledronate (ZA), MgCl_2_ (Mg), both compounds (ZA + Mg) or neither of the two (Ctr). Upper panel shows a bar histogram in which the percentage of CD14 positivity is reported on the y-axis and the tested treatments are indicated on the x-axis. Data are presented as the mean ± S.E.M. values of four independent experiments. Lower panel shows the flow cytometry dot plots obtained in a representative experiment after a double labeling of the CD14 and CD11b antigens. The tested treatments are indicated on the top of each dot plot, whereas percentages of cell subpopulations, exhibiting a differential expression of the two analyzed antigens, are reported inside quadrants. Double positive (CD14+CD11b+) cells are represented in red, whereas all other cell subpopulations are represented in grey.

**Figure 5:**
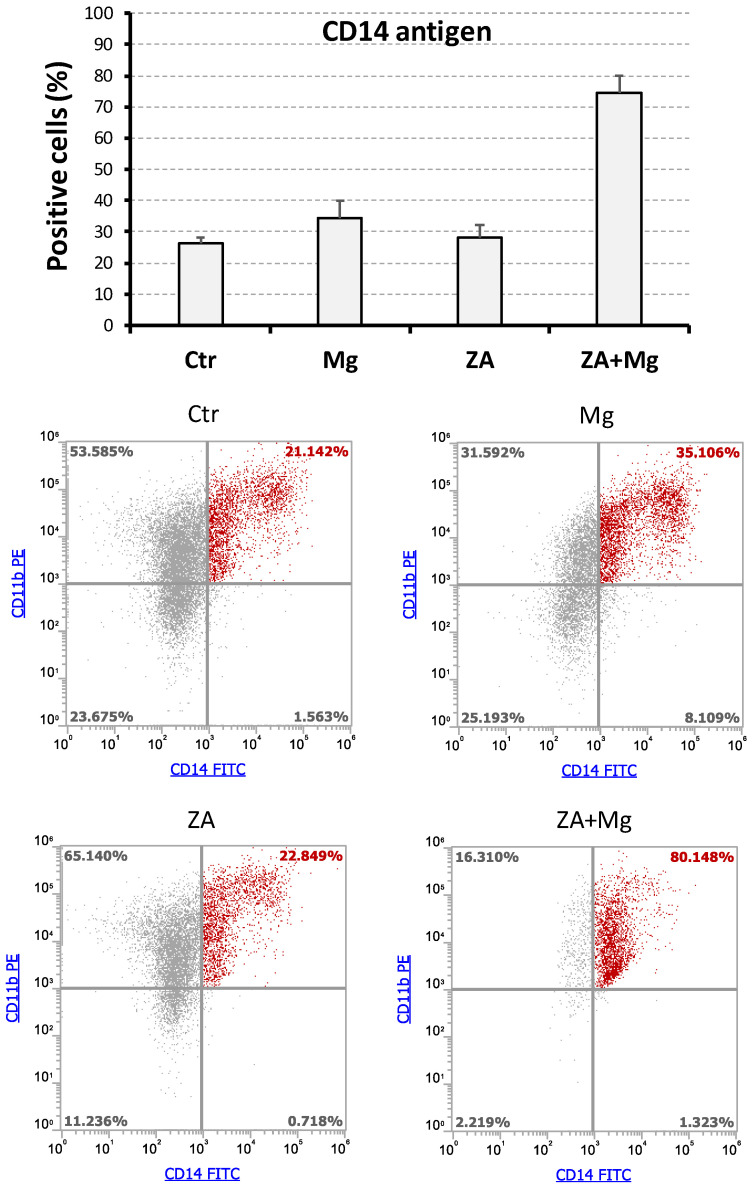
Flow cytometry analysis of surface antigens in THP-1-derived osteoclasts after a 14-day stimulation with M-CSF/RANKL and a parallel treatment with ZA/MgCl_2_. Expression of CD14 and CD11b surface antigens was assessed by flow cytometry in THP-1 cells differentiated to osteoclasts upon a 2-day incubation with PMA followed by 14-day stimulation with M-CSF and RANKL. Treatment conditions, analysis modalities, and data presentation are the same as in Figure 4.

To better characterize the effects determined by treatment with MgCl_2_ and ZA on the osteoclast differentiation of THP-1 cells, we conducted flow cytometry analysis of CD14 and CD11b surface antigens that were detected using a double labeling approach, as already described [53]. For this purpose, THP-1 underwent a 2-day stimulation with PMA followed by a 7-day and 14-day stimulation with M-CSF and RANKL. Immune phenotype was then assessed at both timings, measuring the positivity percentage of the studied antigens. The results of these experiments are, respectively, presented in Figure 4 and Figure 5.

In general, virtually all cells at day 7 and the large majority at day 14 post-stimulation demonstrated the ability to express the CD11b antigen, with minimal variations among the different treatment conditions. Moreover, all CD14+ cells appeared also as CD11b+ and thus CD14+CD11b+ or, in other terms, double positive. Consequently, for simplicity, we preferred to use the percentage of CD14+ cells, rather than CD14+CD11b+, to describe the results of this set of experiments. At day 7, the highest value of such parameter was observed in cells receiving combined treatment with ZA and MgCl_2_, with values a little lower in the other samples (Figure 4). At day 14, we observed a decrease in CD14-positive percentage in almost all samples, with the only exception, again, of cells co-treated with the compounds under analysis, which maintained expression levels comparable to those of day 7 disclosing, at the same time, a synergic interaction between ZA and MgCl_2_ (Figure 5). An example of this finding is provided by a representative experiment, performed at day 14, in which cells co-treated with ZA and MgCl_2_ exhibited 80% of CD14 positivity against 21% of control cells, 35% of MgCl_2_-, and 23% of ZA-treated cells. Notwithstanding, this sample exhibited a lower fluorescence intensity in comparison with the other samples at day 14, and even with the same sample at day 7. Although the immune phenotype does not allow us to distinguish between macrophages and osteoclasts, it has to be pointed out that this decline in CD14 antigen expression is typical of osteoclast differentiation [59,60]. The partial discrepancy between the expression profile of CD14 mRNA (Figure 2) and its corresponding protein, especially observed at day 7 (Figure 4), in our opinion, could be interpreted as the result of different down-regulation kinetics of membrane protein, likely more stable and characterized by a long half-life, in comparison with its mRNA, having opposite properties. The ANOVA p-value of CD14 positivity was highly statistically significant for both the considered timings, and this observation was also confirmed by the Bonferroni test between ZA + MgCl_2_ treatment and ZA treatment (Table S4, Supplementary Material). Interestingly, the presence of a CD14+CD11b- cell fraction appeared to be specifically confined to cells treated with MgCl_2_, where it could be quantified around 3% at day 7 and 8% at day 14. The biological nature of this small cell population is unknown and remains to be clarified.

### 3.4. Morphological and Cytochemical Changes Determined by Treatment with MgCl_2_ and ZA During the Osteoclast Differentiation of THP-1 Cells, Induced by Stimulation with Phorbol Esters, M-CSF, and RANKL

To provide a more complete characterization of the effects determined on osteoclast differentiation by treatment with MgCl_2_ and ZA, THP-1 cells under the same experimental conditions of the previous paragraph were analyzed for their morphological aspect and TRAP activity. Morphological assessment was conducted at day 7 post-stimulation by inverted microscope observation of live images directly obtained from cell cultures, whereas the TRAP assay was carried out on cyto-centrifuged specimens at day 14 post-stimulation followed by microscope examination, as already suggested [53].

**Figure 6:**
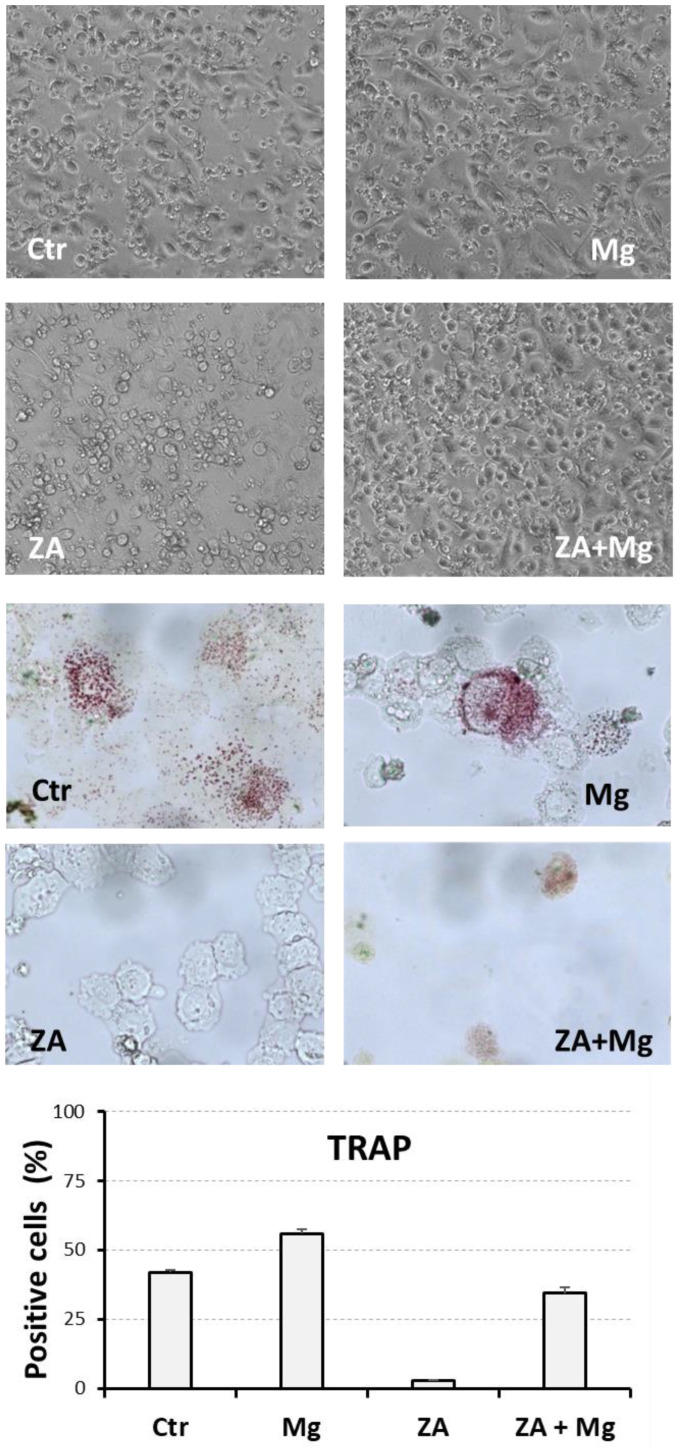
Morphological and cytochemical analysis of THP1-derived osteoclasts after a 7- to 14-day stimulation with M-CSF/RANKL and parallel treatment with ZA/MgCl_2_. Cell samples under the experimental conditions already described in the legend of Figure 4 and Figure 5 were subjected to inverted microscope visualization of cell culture plates (at day 7, upper panel), and TRAP assay on cyto-centrifuged slides followed by microscope examination (at day 14, middle panel). The results of the TRAP assay are also presented as a bar histogram showing the percentage of positive cells (lower panel). Tested treatments are indicated inside each picture using the same acronyms of previous figures.

The results of these analyses are presented in Figure 6, where the upper panel shows, in particular, that control cells and cells treated with MgCl_2_ exhibit a morphology consistent with a macrophage/osteoclast nature, characterized by the presence of larger, polygonal, adhering cells, while cells treated with ZA appeared smaller, round, and partially detached from the substrate, compatibly with an arrested differentiation and apoptotic behavior. Cells subjected to combined treatment with ZA and MgCl_2_, on the other hand, displayed a morphology resembling that of control or MgCl_2_-treated cells, suggesting that MgCl_2_ is able to hinder the biological effects determined by ZA. The middle panel of the same figure shows that TRAP activity, testified by the presence of red staining at the cellular level, can be clearly visualized in control and MgCl_2_-treated cells, while it is undetectable in ZA-treated cells and rescued by the combined treatment with ZA and MgCl_2_. This observation was confirmed by the quantitative count of TRAP-positive cells which averaged 42% in control cells, 56% in MgCl_2_-treated cells, 3% in ZA-treated cells, and 34% in cells co-treated with ZA and MgCl_2_ (lower panel). Similarly, the mean numbers of TRAP-positive cells per mm^2^ resulted, respectively, in 646, 1083, 52, and 521, appearing comparable to those observed by Wu L. et al. performing a similar experiment in which normal monocytes were treated with supra-physiological concentrations of MgCl_2_ [43]. The ANOVA p-value, calculated on the percentage of TRAP-positive cells, were highly statistically significant, and this observation was also confirmed by the Bonferroni test between ZA + MgCl_2_-treated and ZA-treated cells (Table S5, Supplementary Material). The same analysis was, instead, only statistically significant on the absolute number of TRAP-positive cells/mm^2^ (Table S6, Supplementary Material), indicating the capacity of the cell percentage to avoid the effects of a casual variation in the total cell number among different experiments. It is worth underlining that the results of the TRAP assay mimicked almost perfectly the mRNA expression data obtained by the QRT-PCR analysis of the APC5 gene, presented in Figure 1, which exactly codes for the enzyme analyzed in this assay.

## 4. Discussion

Anti-resorptive agents are used to cure a number of metabolic and neoplastic diseases that are all characterized as common features by the loss of bone tissue. The pharmacological activity of these drugs is substantially mediated by their capacity to determine a systemic impairment of osteoclast number. In the case of BPs, this effect can be mainly ascribed to the induction of osteoclast apoptosis [9], whereas DMab, thanks to its anti-RANKL property, is rather responsible for a decreased production of osteoclasts [10]. Regardless of the drug category and mechanism of action, these agents are able to provoke a frightening side effect named ONJ [13,14,15], which is characterized by the infection, inflammation, and necrosis of maxillary or mandibular bone, often triggered by a tooth extraction [19,20]. Based on these premises, it is plausible to hypothesize that an approach able to restore osteoclast number in affected bone might prevent or even cure ONJ. Our previous work in the field under discussion has suggested that this role could be played by a topical use of MgCl_2_ [33].

The role of magnesium on osteoclast differentiation is highly debated in the literature. In particular, the effects of magnesium excess on osteoclasts are influenced by various factors like the formulation used (metal or salt) and the species considered (rodents or humans). Several studies have shown that hyper-stimulation with magnesium inhibits osteoclast activity in rats [37,38], whereas in humans, it has been observed to promote activation [39,40]. Additionally, metallic magnesium has demonstrated an ability to inhibit osteoclastogenesis [41,42], while magnesium salts tend to enhance it [39,40]. These opposing effects have also been confirmed within the context of a single study, where these two distinct magnesium formulations were incubated with cultured osteoclasts derived from human primary monocytes [43]. Experiments conducted in our laboratory have demonstrated that supra-physiological concentrations of MgCl_2_ promote osteoclast differentiation in a human vitamin D3-dependent model of osteoclast differentiation, based on the U937 cell line. To confirm these results also in a RANKL-dependent model of osteoclastogenesis, we used a human cell line called THP-1, which has the characteristic of differentiating into osteoclasts upon a 2-day stimulation with PMA, followed by another 7- to 14-day stimulation with M-CSF and RANKL. Pharmacological treatments were then carried out as already explained for U937 cells [33] using the same compounds (MgCl_2_ and ZA alone or together) at the same concentrations and for the entire duration of experiment (see also Results for more details). This model seems to be quite reliable, since the administration of ZA confirmed its pro-apoptotic effect and its ability to inhibit osteoclast differentiation. The evaluation of the effects promoted by different treatments on osteoclast differentiation was based on molecular, immune phenotype, morphological, and functional (TRAP assay) analysis.

The results of molecular analysis showed that treatment with MgCl_2_ alone induced the expression of six out of the eight differentiation markers analyzed (RANK, CTSK, MMP9, MAFB, CD14, and CD163), while the remaining two (NFATC1, ACP5) remained unchanged compared to the control. Treatment with ZA alone caused a pronounced inhibition of mRNA expression for five of the eight differentiation markers (RANK, ACP5, MMP9, CD14, and CD163), while it had no effect on the remaining three (NFATC1, CTSK, MAFB). The addition of MgCl_2_ to ZA counteracted the inhibitory effect of ZA, leading to transcript levels that were close to (ACP5), overlapping with (MMP9, CD14, CD163), or greater (RANK, NFATC1, CTSK, MAFB) than those observed in the control. Furthermore, for a couple of these genes, specifically NFATC1 and CTSK, a synergic effect between MgCl_2_ and ZA was detected. The immune phenotype analysis, morphological examination, and TRAP assay confirmed molecular data, globally indicating that treatment with MgCl_2_ is able, in all the considered circumstances, to contrast the inhibitory effect exerted by ZA on the differentiation of THP-1 cells. Of note, a synergism between MgCl_2_ and ZA was again observed in the up-regulation of the CD14 surface antigen, assessed by flow cytometry analysis. An aspect of our results deserving some attention is that MgCl_2_ promoted a mixed macrophage/osteoclast differentiation in THP-1 cells but, in light of the finality of our study, we do not think that this observation should be considered a disadvantage. In fact, besides being the immediate precursors of osteoclasts, macrophages are inhibited by ZA exactly as osteoclasts [61], and this finding was also confirmed by the data presented here (see Results). Furthermore, this inhibition has been demonstrated to contribute to ONJ development [62] and, similarly, it is generally believed that the rescue of macrophage function could favor to the recovery from such a condition [63]. From this point of view, the THP-1 cell line appears suitable in an in vitro model to study the role played by macrophages and osteoclasts in ONJ. Although not exactly identical and, in some respects, less striking, the data obtained in THP-1 cells essentially confirmed the results previously observed in U937 cells. On the other hand, the differences observed between the two sets of experiments can reasonably be attributed to the two distinct biological systems under comparison: vitamin D3-dependent osteoclastogenesis in one case and RANKL-dependent osteoclastogenesis in the other. In this regard, it is worth considering that, at least at the molecular level, MgCl_2_ appeared to be more effective in the presence of vitamin D3. Globally, however, the ability of MgCl_2_ to promote osteoclast differentiation, both in the absence and presence of ZA, was once again demonstrated, suggesting a potential therapeutic application of our finding in both the BP- and DMab-induced forms of ONJ.

In order to undergo a real clinical application, of course, this conclusion needs to be properly validated, in vitro, on osteoclasts derived from normal primary monocytes, and in vivo, in patients affected by ONJ. For this purpose, osteoclasts derived from THP-1 cells and normal monocytes, due to their common RANKL dependence, could help perform dose–response experiments with different concentrations of MgCl_2_ and RANKL, aimed to provide a more precise titration of their relative biological activities. This would especially aid to support the biological rationale underlying the use of topical MgCl_2_ in the therapy of the DMab-induced form of ONJ.

Further investigation is also necessary to investigate the mechanism underlying the MgCl_2_-driven stimulation of osteoclast differentiation. Although this mechanism is substantially unknown, we have previously hypothesized that the osteoclast-inducing activity of magnesium could rely in the fact that an excess of its ion version (Mg^2+^) could promote the formation of Mg_2_ATP, in place of MgATP^2−^, leading to the inactivation of some target enzyme normally inhibiting osteoclast differentiation [33]. The described succession of events would, therefore, finally result in the potentiation of this process. In addition, the capacity of this chemical element to counteract the inhibitory effect determined by ZA on osteoclast differentiation, sometimes even resulting in a synergic interaction between the two, might be due to the fact that, always in its ion form (Mg^2+^), it is required for ZA and also for its normal counterpart pyrophosphate (PP) to bind the active site of its FPPS target enzyme. An excess of Mg^2+^ could consequently favor PP in competition with its analog ZA, or perhaps also with other BPs [33]. Obviously, future and specifically designed gene function studies are necessary to verify these hypotheses.

## 5. Conclusions

The conclusions of our work are that, in addition to the ability to support osteoclast differentiation under treatment with ZA in vitamin D3-dependent osteoclastogenesis, MgCl_2_ is also capable of inducing a similar effect in RANKL-dependent osteoclastogenesis, suggesting that a topical therapy based on this compound, in addition to BP-induced ONJ, might also be useful in the cure of ONJ caused by treatment with DMab.

## 6. Patents

The results presented in this manuscript have been previously used to file the International Patent PCT/IB2024/051172 entitled “Prevention and treatment of maxillary osteonecrosis” on 08/02/2024.

## Figures and Tables

**Table 1:** Numerical values obtained by the QRT-PCR analysis of differentiation markers in THP-1-derived osteoclasts under treatment with ZA and MgCl_2_. Table showing the numerical values of the QRT-PCR results presented in Figure 1 and Figure 2. Transcript levels are indicated as the mean ± S.E.M. of relative quantity values obtained in four independent experiments.

Analyzed Marker	Ctr	Mg	ZA	ZA + Mg
RANK	1	1.7 ± 0.2	0.7 ± 0.1	1.7 ± 0.1
NFATC1	1	1.0 ± 0.1	1.2 ± 0.1	1.4 ± 0.3
ACP5	1	1.0 ± 0.1	0.4 ± 0.1	0.7 ± 0.1
CTSK	1	1.4 ± 0.1	1.1 ± 0.1	2.1 ± 0.3
MMP9	1	3.2 ± 0.4	0.3 ± 0.1	1.1 ± 0.1
MAFB	1	1.9 ± 0.1	0.8 ± 0.1	1.8 ± 0.2
CD14	1	2.2 ± 0.3	0.5 ± 0.1	1.0 ± 0.2
CD163	1	3.1 ± 0.4	0.5 ± 0.1	1.2 ± 0.1

**Table 2:** Numerical values and statistical data obtained by flow cytometry analysis of apoptosis and cell cycle in THP-1-derived osteoclasts under treatment with ZA and MgCl_2_. Table showing the numerical values of the flow cytometry results presented in Figure 3. The percentage of apoptotic cells and cells distributed in the various phases of the cell cycle are indicated as the mean ± S.E.M. values obtained in four independent experiments.

Analyzed Parameter	Ctr	Mg	ZA	ZA + Mg
Apoptosis	26.1 ± 3.9	21.5 ± 3.9	37.5 ± 5.7	41.6 ± 3.0
G0/G1	71.7 ± 1.3	62.4 ± 3.0	64.1 ± 2.7	58.1 ± 1.6
S	12.8 ± 1.1	15.0 ± 1.6	18.0 ± 1.8	18.4 ± 1.8
G2/M	15.5 ± 0.5	22.6 ± 2.5	17.9 ± 1.3	23.5 ± 1.9

## Data Availability

The data presented in this study are available on request from the corresponding author.

## Supplementary Materials

The following supporting information can be downloaded at: https://www.mdpi.com/article/10.3390/biology14050533/s1, Table S1: statistical analysis of the data showed in Figure 1, Figure 2 and Table 1; Table S2: statistical analysis of the data showed in Figure 3 upper panel and Table 2; Table S3: statistical analysis of the data showed in Figure 3 lower panel; Table S4: statistical analysis of the data showed in Figure 4 and Figure 5; Tables S5 and S6: statistical analysis of the data showed in Figure 6 lower panel.
