# Supplementary material for: Supra-Physiological Levels of Magnesium Counteract the Inhibitory Effect of Zoledronate on RANKL-Dependent Osteoclastogenesis
Source: Biology (Basel). 2025 May 11;14(5):533. doi: 10.3390/biology14050533 (PMC12109320; doi:10.3390/biology14050533)
Supplement: Supplementary file 1 [file biology-14-00533-s001.zip › Table S1, supplementary material.pdf]

Table S1, Supplementary Material

Analyzed Marker	Ctr	Mg	ZA	ZA + Mg	ANOVA	Bonferroni's multiple comparisons test					
						Mg vs Ctr	ZA vs Ctr	Mg vs ZA	ZA+Mg vs Ctr	ZA+Mg vs Mg	ZA+Mg vs ZA
					p value	Adjusted p value	Adjusted p value	Adjusted p value	Adjusted p value	Adjusted p value	Adjusted p value
**RANK**	1	1.7 ± 0.2	0.7 ± 0.1	1.7 ± 0.1	**0.000044**	**0.007202**	0.389797	**0.000153**	**0.012877**	1	**0.000257**
**NFATC1**	1	1.0 ± 0.1	1.2 ± 0.1	1.4 ± 0.3	0.122536	1	1	1	0.257825	0.217516	1
**ACP5**	1	1.0 ± 0.1	0.4 ± 0.1	0.7 ± 0.1	**0.000001**	1	**0.000006**	**0.000003**	**0.003257**	**0.001425**	**0.017738**
**CTSK**	1	1.4 ± 0.1	1.1 ± 0.1	2.1 ± 0.3	**0.000191**	0.307608	1	0.888244	**0.000264**	**0.017152**	**0.000808**
**MMP9**	1	3.2 ± 0.4	0.3 ± 0.1	1.1 ± 0.1	**<0.000001**	**0.000025**	0.281789	**<0.000001**	1	**0.000035**	0.187653
**MAFB**	1	1.9 ± 0.1	0.8 ± 0.1	1.8 ± 0.2	**0.000061**	**0.000919**	1	**0.000236**	**0.002172**	1	**0.000502**
**CD14**	1	2.2 ± 0.3	0.5 ± 0.1	1.0 ± 0.2	**0.000263**	**0.003397**	0.317533	**0.00024**	1	**0.003397**	0.317533
**CD163**	1	3.1 ± 0.4	0.5 ± 0.1	1.2 ± 0.1	**0.000005**	**0.000038**	0.294106	**0.000006**	1	**0.000071**	0.09629
