# Supplementary material for: Supra-Physiological Levels of Magnesium Counteract the Inhibitory Effect of Zoledronate on RANKL-Dependent Osteoclastogenesis
Source: Biology (Basel). 2025 May 11;14(5):533. doi: 10.3390/biology14050533 (PMC12109320; doi:10.3390/biology14050533)
Supplement: Supplementary file 1 [file biology-14-00533-s001.zip › Table S2, S3, supplementary material.pdf]

Table S2, Supplementary Material

Analyzed Marker	Ctr	Mg	ZA	ZA + Mg	ANOVA	Bonferroni's multiple comparisons test					
						Mg vs Ctr	ZA vs Ctr	Mg vs ZA	ZA+Mg vs Ctr	ZA+Mg vs Mg	ZA+Mg vs ZA
					p value	Adjusted p value	Adjusted p value	Adjusted p value	Adjusted p value	Adjusted p value	Adjusted p value
**Apoptosis**	26.1 ± 3.9	21.5 ± 3.9	37.5 ± 5.7	41.6 ± 3.0	**0.000222**	0.905056	**0.022014**	**0.002399**	**0.003001**	**0.000443**	1
**G0/G1**	71.7 ± 1.3	62.4 ± 3.0	64.1 ± 2.7	58.1 ± 1.6	**0.025694**	0.17372	0.372585	1	**0.025334**	1	0.775555
**S**	12.8 ± 1.1	15.0 ± 1.6	18.0 ± 1.8	18.4 ± 1.8	**0.048633**	1	0.134017	0.864579	0.09724	0.635805	1
**G2/M**	15.5 ± 0.5	22.6 ± 2.5	17.9 ± 1.3	23.5 ± 1.9	**0.008586**	**0.03618**	1	0.252666	**0.018101**	1	0.120688

Table S3, Supplementary Material

Analyzed Marker	Ctr	Mg	ZA	ZA + Mg	ANOVA	Bonferroni's multiple comparisons test					
						Mg vs Ctr	ZA vs Ctr	Mg vs ZA	ZA+Mg vs Ctr	ZA+Mg vs Mg	ZA+Mg vs ZA
					p value	Adjusted p value	Adjusted p value	Adjusted p value	Adjusted p value	Adjusted p value	Adjusted p value
**p21**	1	1.7 ± 0.1	1.3 ± 0.1	2.5 ±0.5	**0.014985**	0.717265	1	1	**0.017694**	0.27498	0.060801
