# Supplementary material for: Supra-Physiological Levels of Magnesium Counteract the Inhibitory Effect of Zoledronate on RANKL-Dependent Osteoclastogenesis
Source: Biology (Basel). 2025 May 11;14(5):533. doi: 10.3390/biology14050533 (PMC12109320; doi:10.3390/biology14050533)
Supplement: Supplementary file 1 [file biology-14-00533-s001.zip › Table S4, S5, S6, supplementary material.pdf]

Table S4, Supplementary Material

Analyzed Marker<br><br>CD14+ cells	Ctr	Mg	ZA	ZA + Mg	ANOVA	Bonferroni's multiple comparisons test					
						Mg vs Ctr	ZA vs Ctr	Mg vs ZA	ZA+Mg vs Ctr	ZA+Mg vs Mg	ZA+Mg vs ZA
					p value	Adjusted p value	Adjusted p value	Adjusted p value	Adjusted p value	Adjusted p value	Adjusted p value
**Day 7**	56.1 ± 3.2	68.9 ± 4.2	71.7 ± 4.5	84.6 ± 2.9	**0.000001**	**0.000668**	**0.000146**	1	**<0.000001**	**0.000144**	**0.000658**
**Day 14**	26.2 ± 2.0	34.5 ± 5.4	28.0 ± 4.0	74.4 ± 5.8	**0.000236**	1	1	1	**0.000483**	**0.001957**	**0.000643**

Table S5, Supplementary Material

Analyzed Marker	Ctr	Mg	ZA	ZA + Mg	ANOVA	Bonferroni's multiple comparisons test					
						Mg vs Ctr	ZA vs Ctr	Mg vs ZA	ZA+Mg vs Ctr	ZA+Mg vs Mg	ZA+Mg vs ZA
					p value	Adjusted p value	Adjusted p value	Adjusted p value	Adjusted p value	Adjusted p value	Adjusted p value
**TRAP+ cells (%)**	42.0 ± 0.6	56.0 ± 1.5	2.7 ± 0.3	34.3 ± 2.3	**0.000002**	**0.004853**	**0.000014**	**0.000002**	0.087263	**0.000438**	**0.000049**

Table S6, Supplementary Material

Analyzed Marker	Ctr	Mg	ZA	ZA + Mg	ANOVA	Bonferroni's multiple comparisons test					
						Mg vs Ctr	ZA vs Ctr	Mg vs ZA	ZA+Mg vs Ctr	ZA+Mg vs Mg	ZA+Mg vs ZA
					p value	Adjusted p value	Adjusted p value	Adjusted p value	Adjusted p value	Adjusted p value	Adjusted p value

**TRAP+ cells/mm²**	645.8 ± 240.3	1083.3 ± 192.9	52.1 ± 10.4	520.8 ± 180.7	**0.033399**	0.780847	0.328997	**0.036792**	1	0.390540	0.656509
